# Effects of Labrador Tea, Peppermint, and Winter Savory Essential Oils on *Fusobacterium nucleatum*

**DOI:** 10.3390/antibiotics9110794

**Published:** 2020-11-10

**Authors:** Amel Ben Lagha, Katy Vaillancourt, Patricia Maquera Huacho, Daniel Grenier

**Affiliations:** Oral Ecology Research Group, Faculty of Dentistry, Université Laval, Québec City, QC G1V 0A6, Canada; amelbenlagha@gmail.com (A.B.L.); katy.Vaillancourt@greb.ulaval.ca (K.V.); patricia_mmaquera@hotmail.com (P.M.H.)

**Keywords:** biofilm, essential oil, *Fusobacterium nucleatum*, halitosis, oral keratinocytes, volatile sulfur compounds

## Abstract

Bad breath or halitosis is an oral condition caused by volatile sulfur compounds (VSC) produced by bacteria found in the dental and tongue biofilms. *Fusobacterium nucleatum* is a Gram-negative anaerobic bacterium that has been strongly associated with halitosis. In this study, essential oils (EO) from three plants, Labrador tea (*Rhododendron groenlandicum* [Oeder] Kron & Judd), peppermint (*Mentha* x *piperita* L.), and winter savory (*Satureja montana* L.), were investigated for their effects on growth, biofilm formation and killing, and VSC production by *F. nucleatum*. Moreover, their biocompatibility with oral keratinocytes was investigated. Using a broth microdilution assay, winter savory EO and to a lesser extent Labrador tea and peppermint EO showed antibacterial activity against *F. nucleatum*. A treatment of pre-formed biofilms of *F. nucleatum* with EO also significantly decreased bacterial viability as determined by a luminescence assay monitoring adenosine triphosphate production. The EO were found to permeabilize the bacterial cell membrane, suggesting that it represents the target of the tested EO. The three EO under investigation were able to dose-dependently reduce VSC production by *F. nucleatum*. Lastly, no significant loss of cell viability was observed when oral keratinocytes were treated with the EO at concentrations effective against *F. nucleatum*. This study supports the potential of Labrador tea, peppermint, and winter savory EO as promising agents to control halitosis and promote oral health.

## 1. Introduction

Bad breath or oral malodor emanating from the oral cavity is commonly known as halitosis and it is considered a multifactorial disease. On the one hand, the main oral sources of halitosis are usually related to tongue coating and periodontal diseases [1,2]. On the other hand, extra-oral sources of halitosis such as disturbances of the upper and lower respiratory tracts, systematic diseases, as well as metabolic changes and renal insufficiencies, also have been reported [3]. Among the major substances related to oral halitosis, volatile sulfur compounds (VSC), including hydrogen sulfide (H_2_S) and methyl mercaptan (CH_3_SH), have been reported to be end-products of protein metabolism in oral bacteria [4].

Gram-negative anaerobic bacteria that have been associated with periodontal disease are important producers of VSC [4,5,6]. More specifically, *Fusobacterium nucleatum* produces large amounts of VSC and is considered a representative bacterial species in halitosis [6,7]. *F. nucleatum* is an indigenous member of the oral microbiome and plays a central role in periodontal biofilm maturation through its ability to act as a bridge between the early and late colonizers in oral biofilms [8]. In addition to playing a role in periodontal disease, *F. nucleatum* has been associated with clinical infections, including appendicitis, osteomyelitis, atherosclerosis, pericarditis, and brain abscesses [9]. Over the last decade, evidence has been presented to support a role of *F. nucleatum* in preterm/low birth weight and colorectal cancer [9,10].

To reduce the amount of halitosis-causing bacteria in the oral cavity, mechanical methods, including tooth brushing and tongue cleaning, are used [11]. In addition, chemical methods such as the use of mouthwashes containing antimicrobials are among the strategies available to reduce both halitosis-causing bacteria and VSC [11]. Traditional medicinal plants represent an attractive source of molecules for the development of new safe agents controlling halitosis-causing bacteria. More specifically, essential oils (EO) are isolated from aromatic plants and have been shown to possess various therapeutic benefits such as anti-inflammatory, antioxidant, and antibacterial properties [12,13]. Preliminary assays performed in our laboratory identified the EO from Labrador tea (*Rhododendron groenlandicum* [Oeder] Kron & Judd) [14], peppermint (*Mentha* x *piperita* L.) [15], and winter savory (*Satureja montana* L.) [16] as highly effective against oral bacterial pathogens. The aim of this study was to investigate the effects of these three EO on growth, biofilm formation and killing, as well as VSC production in *F. nucleatum*. Moreover, the biocompatibility of the EO with oral keratinocytes was assessed.

## 2. Results

Table 1 reports the chemical composition in volatile components of the three EO used in this study. The most predominant components in the Labrador tea EO were α- and β-selinene (19.8%), sabinene (11.9%), germacrene (11.6%), and germacrone (8.5%) The peppermint EO contained mainly menthol (42.3%) and menthone (24.7%). Carvacrol (43.8%), *p*-cymene (14.3%), and γ-terpinene (12.7%) were the major components in the winter savory EO.

The antibacterial activity of Labrador tea, peppermint, and winter savory EO against three strains of *F. nucleatum* was determined using a broth microdilution assay (Table 2). The EO from winter savory had the strongest antibacterial effect with an MIC and MBC in the range of 0.03 and 0.0625% (*v*/*v*), respectively. The Labrador tea EO had an MIC and MBC of 0.25% and 0.5% (*v*/*v*), respectively. Lastly, the peppermint EO had MIC and MBC values in the range of 0.25 and 1% (*v*/*v*), respectively.

**Figure 1:**
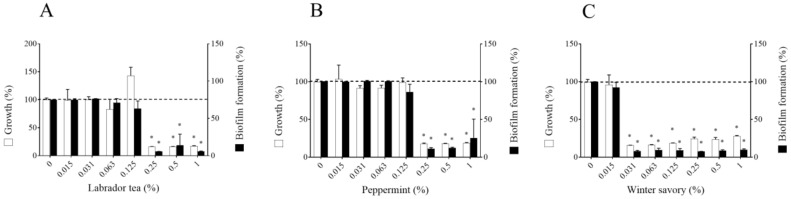
Effect of Labrador tea (**A**), peppermint (**B**), and winter savory (**C**) essential oils on *Fusobacterium nucleatum* ATCC 25586 growth and biofilm formation. Crystal violet staining was used to assess the biofilm biomass. Assays were performed in triplicate, and the means ± standard deviations were calculated. * Significant difference in comparison with the control (no compound) at *p* < 0.01.

Further analyses were performed using *F. nucleatum* ATCC 25586. The three EO were then tested for their effects on biofilm formation by *F. nucleatum*. As reported in Figure 1, they all prevented the formation of biofilm. However, this inhibition was related to an attenuation of bacterial growth caused by the EO.

**Figure 2:**
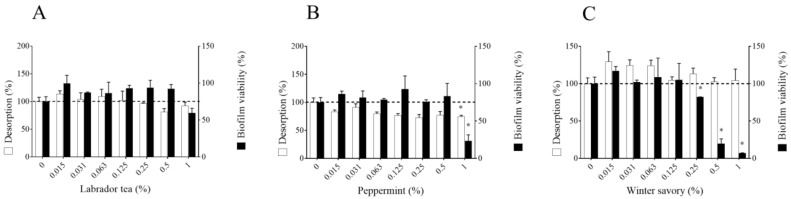
Effect of Labrador tea (**A**), peppermint (**B**), and winter savory (**C**) essential oils on *Fusobacterium nucleatum* ATCC 25586 biofilm desorption and viability. Biofilm desorption was determined by crystal violet staining. Biofilm viability was assessed using a commercial luminescence assay (BacTiter-Glo™) that quantifies ATP, an indicator of metabolically active viable bacteria. Assays were performed in triplicate, and the means ± standard deviations were calculated. * Significant difference in comparison with the control (compound) at *p* < 0.01.

Thereafter, the ability of Labrador tea, peppermint, and winter savory EO to promote biofilm desorption and killing was investigated. No biofilm disruption was observed when pre-formed *F. nucleatum* biofilms were treated (1 h) with different concentrations of Labrador tea and winter savory EO, while the EO from peppermint at 1% (*v*/*v*) induced a slight but significant biofilm desorption (Figure 2). Winter savory (0.25%, 0.5%, and 1% [*v*/*v*]) and peppermint EO (1% [*v*/*v*]) were able to significantly decrease the biofilm viability. More specifically, at 1% (*v*/*v*), EO from winter savory and peppermint decreased viability by 91.8% and 69.1%, respectively. The EO from Labrador tea had no significant effect on the biofilm viability.

**Figure 3:**
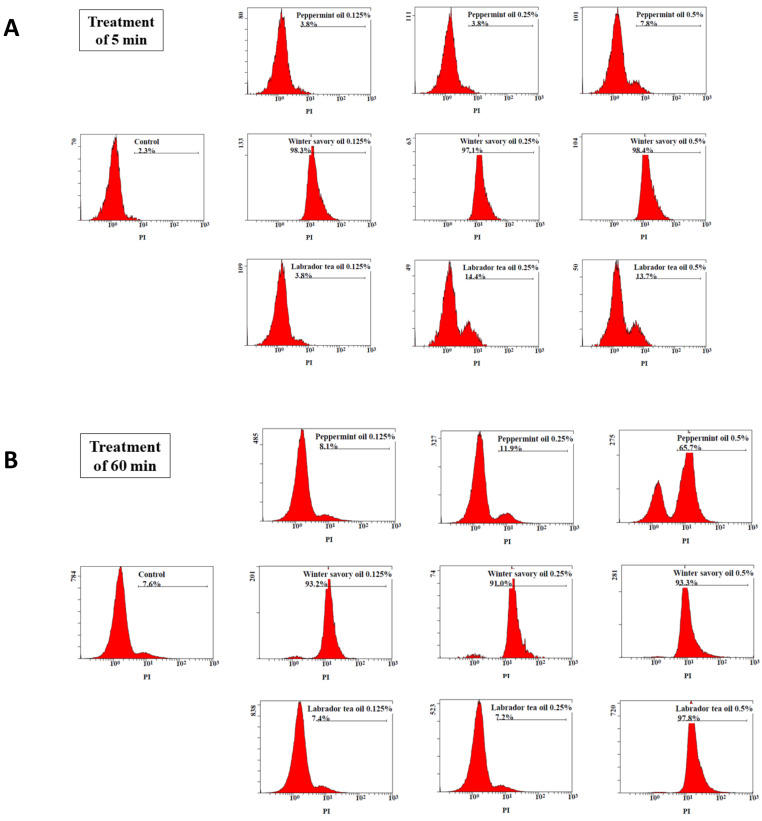
Effect of Labrador tea, peppermint and winter savory essential oils on the membrane permeability of *Fusobacterium nucleatum* ATCC 25586 treated for 5 min (Panel **A**) and 60 min (Panel **B**), as determined by staining DNA with propidium iodide (PI) and flow cytometry analysis. Assays were done in triplicate, and a representative set of data is presented.

The disruption of the cytoplasmic membrane induced by the EO was assessed by using propidium iodide (PI), a fluorescent intercalating DNA dye. Figure 3 reports the % of PI-stained bacteria that corresponds to bacteria with damaged membranes. Untreated *F. nucleatum* (control cells) showed a proportion of PI-positive cells of 2.3% and 7.6% after an incubation of 5 and 60 min, respectively. No marked increase in PI-positive cells (PI-positive cells between: 3.8–13.7%) was observed after a 5-min treatment of bacteria with Labrador tea or peppermint EO at concentrations of 0.125%, 0.25%, and 0.5%. However, extending the treatment to 60 min with Labrador tea and peppermint EO (0.5%) led to an increase of PI-positive cells to 97.8% and 65.7%, respectively. Interestingly, all tested concentrations of winter savory EO (0.125%, 0.25%, and 0.5%) showed a high percentage of PI-positive cells even following a 5-min treatment.

**Figure 4:**
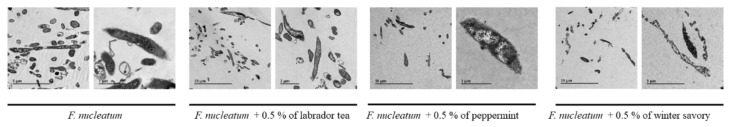
Transmission electron microscopy (TEM) of *Fusobacterium nucleatum* ATCC 25586 cells treated (60 min) with Labrador tea, peppermint, and winter savory essential oils.

Transmission electron microscopy analysis was performed to observe the ultrastructural changes induced by Labrador tea, peppermint, and winter savory EO on *F. nucleatum* following a 60-min treatment (Figure 4). The micrograph of *F. nucleatum* without treatment showed a normal bacillary shape and opacity-electron. Following treatment with the EO, *F. nucleatum* underwent considerable cell alterations. More specifically, membrane breakdown was evidenced for *F. nucleatum* after treatment with peppermint and winter savory EO.

**Figure 5:**
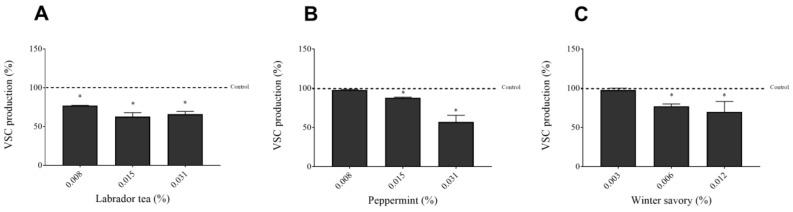
Effect of Labrador tea (**A**), peppermint (**B**), and winter savory (**C**) essential oils on VSC production by *Fusobacterium nucleatum* ATCC 25586. Assays were performed in triplicate, and the means ± standard deviations were calculated. * Significant difference in comparison with the control (no compound; horizontal line) at *p* < 0.01.

The capacity of Labrador tea, peppermint, and winter savory EO to inhibit the production of VSC by *F. nucleatum* was then investigated (Figure 5). All tested concentrations of Labrador tea EO (0.0079%, 0.01563%, and 0.03125%) showed a significant reduction (between 24.3–37.8%) of VSC production. Peppermint and winter savory EO showed a dose-dependent reduction of VCS produced by *F. nucleatum.* Peppermint EO at 0.015% and 0.03125% reduced VCS production by 12.2% and 43.9%, respectively. Winter savory EO at 0.00625 and 0.0125% caused a significant inhibition (between 23.0% and 30.4%) of VSC production.

**Figure 6:**
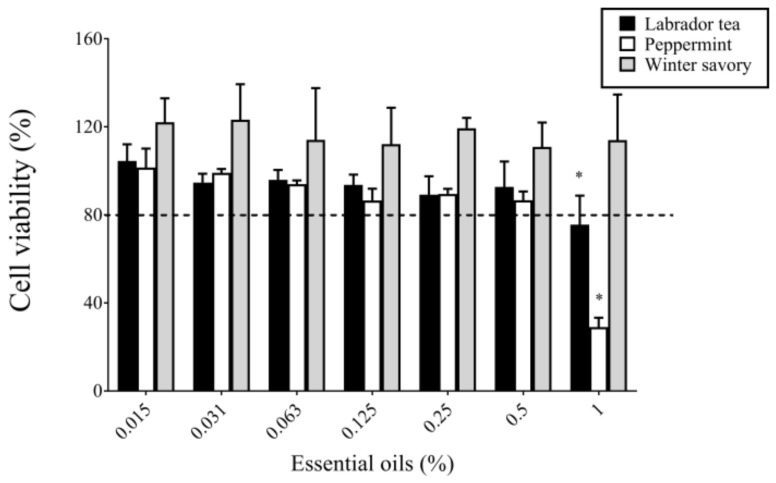
Effect of Labrador tea, peppermint, and winter savory essential oils on the viability of cells of the oral keratinocyte cell line B11 determined using an MTT colorimetric assay. Assays were performed in triplicate, and the means ± standard deviations were calculated. * Significant difference in comparison with the control (no compound; horizontal line) at *p* < 0.01.

Lastly, the cytotoxicity of Labrador tea, peppermint, and winter savory EO was tested on cells of the human oral keratinocyte cell line B11. As shown in Figure 6, all three EO at concentrations ≤0.5% did not show any cytotoxic effects following a 5-min exposition. However, increasing the concentration of Labrador tea and peppermint EO to 1% caused a significant decrease in cell viability.

## 3. Discussion

Many bacterial species within the oral microbiota are involved in the complex process of halitosis. Previous studies have shown an association between periodontopathogenic bacteria and the oral malodor [2]. It has been suggested that Gram-negative anaerobic bacteria are responsible for the main pathway of protein degradation and the metabolism of sulfur-containing amino acids [4]. Among them, *F. nucleatum* has pathogenic potential in periodontal disease and halitosis; the latter is related to its ability to produce large amounts of H_2_S [4,7]. Given its prevalence and importance in the pathogenic process of halitosis and considering its key role in periodontal biofilm maturation, a reduction of *F. nucleatum* could represent a promising therapeutic strategy for controlling oral malodor. In the present study, the ability of Labrador tea, peppermint, and winter savory EO to inhibit growth and biofilm formation, to cause biofilm desorption and killing, and to reduce H_2_S production in *F. nucleatum* was investigated. In view of future clinical applications, the cytotoxicity of the EO on oral keratinocytes was assessed.

Natural antimicrobial agents, such EO derived from several aromatic plants, have been suggested as alternatives for the prevention or treatment of infectious diseases [12,13]. The novelty of this study lies in the fact that the effects of EO from Labrador tea, peppermint, and winter savory on *F. nucleatum* have not been investigated yet. Our results showed that the three EO under investigation were bactericidal against *F. nucleatum*. Winter savory EO, with MIC and MBC values of 0.03125% (*v*/*v*), were found to be significantly more effective than Labrador tea and peppermint EO (MIC = 0.25% and MBC = 0.5%). It is worth mentioning that the three EO tested are likely to present different levels of solubility due to their composition. Therefore, they are not distributed homogeneously in the culture medium and consequently this may lead to imprecision related to serial dilutions. This has to be taken into consideration regarding the MIC and MBC values as well as in the other assays requiring dilutions of the EO. While the antimicrobial activity of winter savory EO against *F. nucleatum* has not been previously reported in the literature, numerous studies showed a strong antimicrobial effect of this EO against human and animal bacterial pathogens, including *Staphylococcus hyicus*, *Staphylococcus aureus*, *Streptococcus suis*, *Actinobacillus pleuropneumoniae*, *Actinobacillus suis*, *Bordetella bronchiseptica*, *Haemophilus parasuis*, *Pasteurella multocida* and *Campylobacter jejuni* [17,18,19,20,21]. 

By using the fluorescent dye propidium iodide and a flow cytometry analysis, evidence was obtained that Labrador tea, peppermint, and winter savory EO exert their antibacterial activity by altering the membrane integrity and permeability of *F. nucleatum*. These results were supported by the transmission electron microscopy observations showing a clear membrane disruption after treatment of *F. nucleatum* with the EO under investigation. Disruption of the bacterial cell membrane affects vital processes such as energy conversion processes, nutrient processing, synthesis of structural macromolecules, and the secretion of growth regulators [22]. Interestingly, the marked antibacterial activity of winter savory EO has been related to the ability to damage the structure and function of the bacterial membrane [20,23]. 

The ability of EO to interfere with biofilm formation has been previously reported [24,25]. In this study, Labrador tea, peppermint, and winter savory EO attenuated biofilm formation by *F. nucleatum*. However, this effect may not be considered a specific anti-biofilm activity since biofilm reduction appears to be mostly related to growth attenuation. The ability of the EO to induce biofilm desorption and to reduce biofilm viability was also assessed. Our results showed that peppermint EO, but not Labrador tea and winter savory EO, caused a significant desorption of a pre-formed *F. nucleatum* biofilm. However, all three EO were able to reduce the viability of pre-formed *F. nucleatum* biofilms.

*F. nucleatum* is well known to produce high amounts of H_2_S, which is regarded as one of the most common VSC responsible for unpleasant odor [26,27]. Moreover, H_2_S is a metabolite that can modulate inflammatory responses and cause toxic effects in oral epithelial cells and gingival fibroblasts [28,29,30,31]. In a previous study by Pitts et al. [32], it was reported that decreased malodor and VCS are related to the decrease in odor-causing bacteria. Labrador tea, peppermint, and winter savory EO significantly reduced VSC production by *F. nucleatum* as determined by a Halimeter monitor, which mostly monitors H_2_S. One should not exclude the possibility of direct reactions occurring between EO and VSC, thus reducing the amount detected by the Halimeter monitor. Few studies investigated the ability of EO to attenuate VSC production by *F. nucleatum*. Rmaji et al. [33] reported that EO of *Piper betle* L. had an antimicrobial effect against *F. nucleatum* and showed a reduction of VSC production. In a previous study on *Solobacterium moorei*, another oral bacteria associated with halitosis, LeBel et al. reported that cinnamon bark EO inhibited H_2_S production, in addition to exerting a bactericidal effect [34].

Lastly, the cytotoxicity of Labrador tea, peppermint, and winter savory EO towards human oral keratinocytes was analyzed in view of future clinical applications. Oral keratinocytes were selected because they are predominant cells in the oral cavity and represent the first cell type that comes into contact with molecules contained in mouth rinses. The results showed that the EO at concentrations ≤ 0.5% did not cause cytotoxic effects following a 5-min exposure.

The present study showed that Labrador tea, peppermint, and winter savory EO were bactericidal against *F. nucleatum*, planktonic, and biofilm-embedded cells, while having low cytotoxicity against oral keratinocytes. Taking into consideration these data, the three EO tested could be promising compounds to be incorporated into oral hygiene products such toothpastes, mouth rinses or gels for controlling halitosis and periodontal biofilm. Future clinical studies are necessary to evaluate the potential of this strategy.

## 4. Materials and Methods

### 4.1. Bacteria and Growth Conditions

The reference strains *F. nucleatum* ATCC 25586, ATCC 10953, and ATCC 49256 were grown in Todd-Hewitt broth (THB; BBL Microbiology Systems, Mississauga, ON, Canada) supplemented with 0.001% hemin and 0.0001% vitamin K in an anaerobic chamber (80% N_2_, 10% CO_2_, 10% H_2_) at 37 °C.

### 4.2. Essential Oils

EO from Labrador tea (*Rhododendron groenlandicum*; leaf; origin: Canada; lot #: LED12001), peppermint (*Mentha* x *piperita* L.; flowering herb; origin: India; lot #: 1206010), and winter savory (*Satureja montana* L.; flowering top; origin: Spain; lot #: 0908817) were purchased from Hunzaroma Inc. (Longueuil, QC, Canada). The composition the EO, as determined by gas chromatography (GC)-flame ionization detector (FID), and GC-mass spectrometer (MS) analyses and provided by the manufacturer, is given in Table 1.

### 4.3. Determination of Minimum Inhibitory Concentrations and Minimum Bactericidal Concentrations

The minimum inhibitory concentrations (MIC) and minimum bactericidal concentrations of EO against *F. nucleatum* were determined using a broth microdilution assay as routinely performed in our laboratory [19,20]. Briefly, *F. nucleatum* was grown for 24 h, and the culture was diluted in fresh broth medium to an optical density at 660 nm (OD_660_) of 0.2. Equal volumes (100 µL) of bacterial culture and two-fold serial dilutions of EO (from 1% to 0.015%; [*v*/*v*]) in culture medium (overnight pre-incubation under anaerobiosis) were mixed in the wells of a 96-well tissue culture-treated, flat-bottom, microplate (Sarstedt, Newton, NC, USA). Control wells without bacteria or EO were also prepared. The microplate was sealed with a self-adhesive clear polyester film (GE Healthcare, Marlborough, MA, USA). After a 24-h incubation at 37 °C in the anaerobic chamber, bacterial growth was recorded visually and by monitoring the OD_660_. The MIC values of the EO were the lowest concentrations at which no growth occurred. To determine the MBC values, 5-µL aliquots from wells showing no visible growth were spread on culture plates and were incubated for 48 h at 37 °C in the anaerobic chamber. The MBC values of the EO were the lowest concentrations at which no colonies formed. The MIC and MBC assays were done in triplicate, and a representative set of data is presented.

### 4.4. Biofilm Formation

*F. nucleatum* ATCC 25586 was grown in a 96-well microplate as above. After a 48-h incubation under anaerobic conditions, the OD_660_ was recorded prior to removing the planktonic bacteria and spent medium by aspiration using a 26g needle. The biofilm biomass was assessed by crystal violet staining [19,20]. Briefly, the biofilms were stained with 0.01% crystal violet (100 µL) for 15 min. The wells were washed with distilled water to remove unbound crystal violet dye and dried for 2 h at 37 °C. The crystal violet was solubilized by adding 100 µl of 75% (*v*/*v*) ethanol to each well, and the plate was shaken for 15 min prior to measure the absorbance at 550 nm (A_550_) using a Synergy 2 microplate reader (Bio-Tek Instruments, Winooski, VT, USA). Assays were performed in triplicate, and the means ± standard deviations were calculated.

### 4.5. Biofilm Desorption and Killing

The ability of Labrador tea, peppermint, and winter savory EO to promote biofilm desorption and killing was investigated using a previously described procedure [19]. Briefly, 48-h biofilms of *F. nucleatum* ATCC 25586 were pre-formed in a 96-well microplate as above and treated for 1 h with the EO (0.5% to 0.015%; [*v*/*v*]) under anaerobic conditions. A treatment with 50 mM phosphate-buffered saline (PBS), pH 7.2, was used as a control. Afterwards, the biofilms were washed twice with PBS and stained with crystal violet as described above. In addition, the ability of Labrador tea, peppermint, and winter savory EO to decrease the viability of *F. nucleatum* biofilms was also investigated. Pre-formed biofilms of *F. nucleatum* were exposed to EO as above, and the viability was assessed using the commercial luminescence assay (BacTiter-Glo™, Madison, WI, USA) that measures ATP, an indicator of bacterial viability. Luminescence was quantified using a Synergy 2 microplate reader. Assays were performed in triplicate, and the means ± standard deviations were calculated. 

### 4.6. Bacterial Membrane Permeability

The ability of Labrador tea, peppermint, and winter savory EO to permeabilize the cytoplasmic membrane of *F. nucleatum* ATCC 25586 was investigated using propidium iodide, which enters cells with a compromised membrane and reacts with DNA. The protocol of Coronel-Leon et al. [35] with slight modifications was used. Briefly, 50 µl of a stock solution of propidium iodide (1 mg/mL in distilled water) was added to one mL of *F. nucleatum* cells suspended in PBS at an OD_660_ of 0.2 and previously treated (5 and 60 min) with the EO. After 10 min at room temperature, samples were analyzed by flow cytometry using a Cytomics FC500 MPL flow cytometer (Beckman Coulter, Inc., Fullerton, CA, USA). The instrument was set up with the standard red fluorescence (675 nm) configuration. The results were collected on logarithmic scales. Assays were done in triplicate, and a representative set of data is presented.

### 4.7. Transmission Electron Microscopy Observation

Bacterial cells from an overnight culture were washed twice, suspended in PBS at an OD_660_ of 0.5, and incubated in the presence of Labrador tea, peppermint, and winter savory EO (0.5%; [*v*/*v*]) at room temperature for 60 min. Thereafter, bacteria were fixed for 2 h at room temperature in 0.1 M cacodylate buffer (pH 7) containing 5% glutaraldehyde and 0.15% ruthenium red. Cells were then reacted with polycationic ferritin (1 mg/mL) and processed as described by Vanrobaeys et al. [36]. Thin sections were examined using a JEOL 1230 transmission electron microscope at an accelerating voltage of 80 kV. 

### 4.8. VSC Production

*F. nucleatum* ATCC 25586 was grown for 24 h in screw cap tubes in the presence of Labrador tea, peppermint, and winter savory EO (0.0079–0.031%; [*v*/*v*]). Bacterial growth was monitored by recording the OD_660_ prior to measure VSC using a Halimeter sulfide monitor (Interscan Corp. Chatsworth, CA) according to the manufacturer’s protocol. The straw, connected to the monitor, was inserted (2 cm) in the test tube headspace immediately after removing the cap, and the VSC levels were determined as parts per billion (ppb) of sulfide equivalents. The reduction in VSC production caused by the EO tested was calculated by comparison with the control (no EO). Assays were performed in triplicate, and the means ± standard deviations were calculated. 

### 4.9. In Vitro Cytotoxicity

The oral keratinocyte cell line B11 [37], kindly provided by S. Groeger (Justus-Liebig-University Giessen, Germany), was used to evaluate the cytotoxicity of Labrador tea, peppermint, and winter savory EO. Keratinocytes were cultured in keratinocyte serum-free medium (K-SFM; Life Technologies Inc., Burlington, ON, Canada) supplemented with growth factors (50 µg/mL of bovine pituitary extract and 5 ng/mL of human epidermal growth factor) and 100 µg/mL of penicillin G-streptomycin in a humidified incubator with 5% CO_2_ at 37 °C. Keratinocytes (3 × 10^5^ cells) were seeded into wells of a 96-well tissue culture plate and cultivated until confluence. Cells were then treated with the EO (0.015–1% [*v*/*v*]) in complete K-SFM medium for 5 min prior to determine viability using an MTT (3-[4,5-diethylthiazol-2-yl]-2,5diphenyltetrazolium bromide) colorimetric assay (Roche Diagnostics, Laval, QC, Canada). Cells treated with K-SFM medium were used as a control. Assays were performed in triplicate, and the means ± standard deviations were calculated. 

### 4.10. Statistical Analysis

The mean ± standard deviations were analyzed for statistical significance using one-way ANOVA with a post hoc Bonferroni multiple comparison test and were considered significant at *p* < 0.01.

## Figures and Tables

**Table 1:** Main components (in %) identified in Labrador tea, peppermint, and winter savory essential oils. Data from gas chromatography (GC)-flame ionization detector (FID) and GC-mass spectrometer (MS) analyses were provided by the manufacturer (Hunzaroma Inc., Longueuil, QC, Canada).

Components	Labrador Tea	Peppermint	Winter Savory
β-Bisabolene	0.94	-	0.85
Borneol	-	-	2.16
Bornyl acetate	1.89	-	-
Camphene	1.21	-	0.69
Carvacrol	-	-	43.84
β-Caryophyllene	0.6	2.6	-
1,8-Cineol	-	5.31	0.56
*p*-Cymene	1.63	-	14.3
β-Elemene	1.3	-	-
Eudesma-3,7-dien-2-one	1	-	-
Germacrene B	9.76	-	-
Germacrene D	1.82	-	-
Germacrone	8.51	-	-
α-Humulene	2.77	-	-
Ledol	0.81	-	-
Limonene	2.34	2.52	0.5
Linalool	-	-	1.04
Menthofurane	-	7.1	-
Menthol	-	42.3	
Menthone	-	24.7	-
Iso-Menthone	-	3.4	-
Myrcene	-	-	1.11
Myrtenal	2.17	-	-
Oct-1-en-3-ol	-	-	0.64
α-Pinene	3.25	0.76	1.97
β-Pinene	3.25	1.07	-
*trans*-Pinocarveol	1.44	-	-
Pinocarvone	0.83	-	-
Pulegone	-	6.7	-
Sabina ketone	0.5	-	-
Sabinene	11.93	-	-
Dehydro sabinene ketone + *trans*-*p*-mentha-2,8-dien-1-ol	0.65	-	-
α-Selinene	8.89	-	-
β-Selinene	10.95	-	-
Terpinen-4-ol	1.68	-	4.05
α-Terpinene	0.72	-	1.14
γ-Terpinene	2.58	-	12.66
Thuj-3-en-10-al	0.86	-	-
Thymol	-	-	6.71
Thymol methyl ether	-	-	3.09
Different compounds at a concentration < 0.5%	14.12	2.82	3.08

**Table 2:** Minimum inhibitory concentration (MIC) and minimum bactericidal concentration (MBC) values of Labrador tea, peppermint, and winter savory essential oils against *Fusobacterium nucleatum*. Assays were done in triplicate, and a representative set of data is presented.

Essential Oil	MIC (%; *v*/*v*)			MBC (%; *v*/*v*)		
	ATCC 25586	ATCC 10953	ATCC 49256	ATCC 25586	ATCC 10953	ATCC 49256
Labrador tea	0.25	0.25	0.25	0.5	0.5	0.5
Peppermint	0.25	0.5	0.5	0.5	0.5	1
Winter savory	0.031	0.031	0.063	0.031	0.031	0.063
